# Supplementary material for: MetaRibo-Seq measures translation in microbiomes
Source: Nat Commun. 2020 Jun 29;11:3268. doi: 10.1038/s41467-020-17081-z (PMC7324362; doi:10.1038/s41467-020-17081-z)
Supplement: Supplementary file 10 — Supplementary Data 7 [file 41467_2020_17081_MOESM10_ESM.zip › File2/Confidence_High_Taxonomy/97683_out.krona.html]

Javascript must be enabled to view this page.

members
magnitude
magnitudeUnassigned
count
unassigned
taxon
rank

97683\_out

3

2
superkingdom
3

1239
3
phylum

186801
class
3

186802
order
3

1898207

SRS063985\_contig\_number\_contig-100\_3073.165476SRS098571\_contig\_number\_contig-100\_12940.272301
2
species

541000
1
family

genus
1
1263

1265

SRS078419\_contig\_number\_12800
1
species
